# Comparing the Environmental Impacts of Representative Food Donation and Redistribution Strategies

**DOI:** 10.3390/foods15040645

**Published:** 2026-02-11

**Authors:** Zhijian Guo, Tianhong Mu, Beth Feingold, Akiko Hosler, Christine Bozlak, Stacy Pettigrew, Xiaobo Romeiko

**Affiliations:** 1 Department of Mathematics and Statistics, University at Albany, State University of New York, 1400 Washington Avenue, Albany, NY 12222, USA; zguo@albany.edu; 2 Department of Environmental and Sustainable Engineering, University at Albany, State University of New York, 1400 Washington Avenue, Albany, NY 12222, USA; tmu@alumni.albany.edu; 3 Department of Environmental Health Sciences, University at Albany, State University of New York, 1 University Place, Rensselaer, NY 12144, USA; bfeingold@albany.edu; 4 Department of Epidemiology and Biostatistics, University at Albany, State University of New York, 1 University Place, Rensselaer, NY 12144, USA; ahosler@albany.edu; 5 Department of Health Policy, Management, and Behavior, University at Albany, State University of New York, 1 University Place, Rensselaer, NY 12144, USA; cbozlak@albany.edu; 6 Radix Ecological Sustainability Center, 153 Grand Street, Albany, NY 12202, USA; stacy@radixcenter.org

**Keywords:** food donation, life cycle assessment, waste management, supply chain, environmental impacts, greenhouse gas emissions

## Abstract

Assessing the environmental impacts of food donation systems is necessary to support food donation policy and management. Few life cycle assessment (LCA) studies have investigated the environmental impacts of food donation systems. This comparative LCA study analyzed the environmental impacts of eight different donation scenarios reflecting diverse supply chain configurations and operational management options, using 391.8 kg of redistributed food over two weeks as the functional unit. Each of the eight scenarios presented net environmental benefits for all five life cycle environmental impact categories: 132~233 kg CO_2_-eq for global warming potential, 2.30~5.24 kg SO_2_-eq for acidification potential, 1.13~2.04 kg N-eq for eutrophication potential, 1791~3140 MJ for cumulative energy demand, and 3.7 × 10^7^~5.8 × 10^7^ m^3^ for water resource depletion. The highest magnitudes of environmental benefits were achieved when intermediary organizations collected and gleaned the surplus food from donors and then transported it to food pantries (the eighth scenario). Improving the quality of donated food, augmenting the sorting capacities of emergency organizations, and shortening transportation distances could increase the environmental benefits of food donation systems. The environmental impact intensities of production and waste management choices for food waste generated during the redistribution of the surplus food ranked as the top influential factors for the five environmental impacts. Rescuing surplus food from donors who landfilled the wasted food tended to yield larger environmental benefits than from donors who composted it. Overall, this study finds that improving donation quality and increasing the capacities of emergency food organizations are crucial for maximizing the environmental benefits of the fresh produce donation system.

## 1. Introduction

Food donation plays an important role in addressing food waste and food insecurity. In 2010, 30~40% of food was wasted in the United States (US), which was approximately 133 billion pounds and USD 161 billion worth of food [1]. About 20~50% of uneaten fresh vegetables and fruits are edible and nutritious for human consumption [2]. In 2021, around 10% of the population were food insecure in the US [3]. Due to its capability of simultaneously reducing food waste and addressing food insecurity, food donation ranks as one of the top strategies for EPA’s Wasted Food Scale [4]. The emergency food organizations in the US have successfully managed a significantly large amount of food surplus to be delivered to feed people who are experiencing food insecurity. For example, in 2020, the Feeding America Network, through a nationwide network of 200 food banks, rescued 3.6 billion pounds of food and groceries from landfill and provided 4.2 billion meals to food insecure families [5].

The food redistribution practices of emergency food assistance organizations are diverse and require energy and transportation, resulting in different environmental impacts. First, surplus food redistribution has two different sets of redistribution logistics: centralized and decentralized redistribution. Centralized redistribution requires that all donated food goes to the food bank before being distributed to food pantries. Decentralized redistribution allows donated food to be sent directly to food pantries, although the food banks may coordinate the redistribution. Centralized and decentralized redistribution may have different transporting distances of food, subsequently resulting in different emissions. Second, donated food qualities varied, which may lead to different food waste amounts during redistribution. Third, the labor (paid staff or volunteer) availability for sorting food at emergency food organizations influences different waste generation amounts. The Sustainable Development Goals recognize food rescue and redistribution as a valuable strategy for addressing greenhouse gas emissions [6]. However, there is limited data quantifying the environmental benefits of this strategy and how these benefits might vary with different redistribution strategies. This gap limits the ability of emergency food organizations and policymakers to assess the environmental implications of alternative system designs. To design the most environmentally friendly redistribution practices, it is essential to determine and compare the environmental impacts of various strategies.

Life cycle assessment (LCA) is a fundamental approach for systematically estimating resource consumption and the environmental impacts of processes and services [7]. Numerous existing LCA studies have evaluated a range of food waste management practices [8,9,10,11,12], and the majority of these studies mainly focused on the middle and bottom tiers of the food waste hierarchy [13], such as recycling and disposal. An LCA of food donation practices is needed to aid in designing effective alternative strategies for food donation.

To the best of our knowledge, eight existing LCA studies have analyzed the environmental sustainability of food donation systems [14,15,16,17,18,19,20,21]. Despite valuable contributions, there are several knowledge gaps. First, no peer-reviewed study has compared the environmental impacts between centralized and decentralized donated food redistribution strategies in the United States. Six studies [14,15,16,17,18,20] compared the environmental impacts of food donation with food waste management practices such as anaerobic digestion, composting, conversion, incineration, and landfills. One study [19] focused on the environmental impacts of food donation combined with other food waste treatment strategies. Furthermore, in a study by Bergström et al. [21], LCA was employed to assess and compare the environmental impacts of redistributing food surplus via different outlets. We did not find peer-reviewed LCA studies evaluating the influences of distribution logistics, sorting capacity, and food quality on the environmental impacts of surplus food donation and distribution systems. Second, half of these existing eight LCA studies [16,17,18,21] reported only one or two environmental impact categories, which were global warming potential (GWP) and energy or fossil fuel consumption. Only four of these studies [14,15,19,20] reported environmental impact categories of food donation systems covering acidification, eutrophication, and water resource depletion. Since previous food LCAs found significant tradeoffs between GWP and other environmental impact categories [22], more studies are needed on those impact categories of the food donation system. Third, the existing studies considered very limited food species or types in their models. For example, Eriksson et al. [17] and Eriksson & Spångberg [16] selected only five food products as the food surplus in their LCA. Sulis et al. [19] only considered fruit and vegetables as an aggregated group in their study. However, fruits and vegetables’ life cycle environmental impact densities vary significantly, and they are the most desirable food types for the food insecure population [23].

In order to fill in the aforementioned knowledge gaps, this study aimed to quantify and compare the life cycle impacts of various donation/redistribution strategies within the Capital Region of New York State as a case example for 26 types of vegetables and 17 types of fruits. Despite their desirability, vegetables and fruits experience rapid deterioration and constitute a substantial portion of food waste [24]. This study reveals the environmental sustainability of the food donation system and provides a scientific foundation for decision-making in the food donation system.

## 2. Methodology

As suggested by the International Organization for Standardization [25] standard, the LCA includes four phases: goal and scope definition, life cycle inventory analysis, life cycle impact assessment, and life cycle interpretation. The goal and scope phase defines the assessed processes and delineates the boundaries for the supply system. The second phase compiles the life cycle inventory by gathering resource inputs and environmental releases within the defined supply boundary. Following the second phase, the life cycle impact assessment phase translates the life cycle inventory into environmental impacts. The last phase identifies the scenario with the most preferred environmental performance. Additionally, sensitivity analyses are conducted to understand the impacts of data uncertainty on life cycle impacts. The details of each phase are explained below.

### 2.1. Goal and Scope

The goal of this study is to assess the environmental impacts of various representative scenarios for donating and redistributing surplus food implemented by emergency food assistance organizations. The emergency food assistance organizations include food banks, food pantries, and intermediary organizations (such as gleaning organizations with a small capability and service area). Taken together, these are the central actors that connect the food donors and food recipients by redistributing food surplus. This study uses the Capital Region in New York State as a case example. The Capital Region is a mid-sized metropolitan area [population: 1.1 million [26]]. The emergency food assistance organizations in this region, for this study, include the Northeast Regional Food Bank, food pantries, and local gleaners. The Regional Food Bank of Northeastern New York’s service area encompasses 41% of the landmass of New York State [27], and it is the only food bank serving the Capital Region.

**Figure 1:**
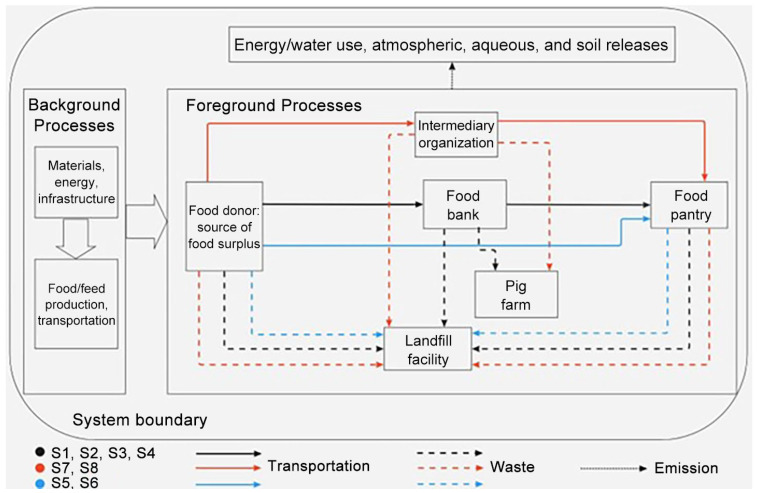
System boundary of life cycle assessment study for surplus food donation and distribution systems.

Figure 1 illustrates the system boundary of this life cycle assessment study for surplus food donation and distribution systems. The system scope included both foreground and background processes. The foreground processes included sourcing food surplus from donors, sorting and redistributing food surplus, and handling food waste. Based on the consultation with emergency food assistance organizations in the Capital Region, the food waste was used as pig feed or composted or disposed of to a landfill. The foreground processes included donors’ on-site food waste management such as composting and necessary transportation among food donors, food banks, and food recipients. The background processes included food/feed, energy, and infrastructure processes, which support the foreground processes. The scope of this study included the energy and water use and associated environmental releases from both foreground and background processes.

The eight scenarios reflect various combinations of supply chain configurations and operational management practices. Scenario 1 (S1) is the baseline scenario and also the current state of practice in food banks, where food surplus is donated and distributed to the food bank and then diverted to food pantries. After the food surplus is donated and transported to the food bank, employees or volunteers at the food bank will check and sort the received food. If the food surplus passes quality control, it is stored and ready for redistribution to food pantries. Otherwise, it is sent to pig farms or landfill facilities. Scenarios 2 (S2), 3 (S3), and 4 (S4) share the same supply chain configurations. Different from S1, S2 has two or more sorters at the food bank to mitigate food waste. More sorters allow for the recovering of more edible foods than less sorters. Different from the previous scenarios, S3 assumes that a higher quality of surplus food is donated to the food bank. S4 is a combination of S2 and S3. Different from the previous four scenarios, Scenarios 5 (S5) and 6 (S6) feature the direct transportation from food donors to food pantries, bypassing the food bank. S6 has a higher quality of donated food surplus, which reduces more food waste than S5. Scenarios 7 (S7) and 8 (S8) include intermediary organizations in the supply chain configurations. For S7, the intermediary organization picks up the food surplus from donors and then delivers the food surplus to the food pantries. For S8, intermediary organizations collect and glean the surplus food from donors and then transport it to food pantries. Due to the gleaning and sorting performed by intermediary organizations, high-quality food enters the redistribution chains and prevents food waste during the redistribution. The above scenarios were suggested and are currently implemented by emergency food assistance organizations.

The functional unit is the reference unit that forms the basis for comparisons among the scenarios. In this study, the weight (391.8 kg) of redistributed food surplus during two weeks’ distribution operation is utilized to be the functional unit for comparing the total life cycle environmental impact of the food surplus redistribution system. This weight-based choice of a functional unit is consistent with the existing literature [20].

### 2.2. Life Cycle Inventory (LCI)

The foreground inventory (Table S2) was based on actual data from the Northeast Regional Food Bank, and the background inventory was compiled based on the state-of-the-art life cycle database ecoinvent v3.10 [28]. The Northeast Regional Food Bank provides over 50 million pounds of food a year [27]. The current state of practice in food banks corresponds to S1 in Table 1. The authors volunteered at the Northeast Regional Food Bank over two weeks in 2019 and obtained records regarding the food types, the weight of redistributed food surplus for each type, the geolocations of donors and recipients, and transportation vehicle types for donation and redistribution. Forty-three types of fresh produce were distributed, and their associated weight shares are summarized in Table 2. During the two weeks of the on-site observation period, the amount of food received by the food bank was 391.8 kg, and the amount of food distributed by the food bank was 217.7 kg. Based upon these values, the waste portion during the collecting and redistribution of food surplus in S1 was around 55%. Based on the on-site observations, 30% more products can be recovered and redistributed for human consumption when two or more sorters were added at the food bank in S2. Schupp et al.’s study also revealed that the sorting effort reduced food waste rate by 30.5% on average in the lunchroom of a Washington state school district [29]. When a higher quality of food entered the donation and redistribution chain, less food waste occurred at the quality check point of the food bank in S3. Through consultations with community partners, we considered 30% less food waste in S3 than S1. S4 presents improvements from both S2 and S3. For S5, 30% of food surplus was assumed to become waste during the redistribution chain. The value of 30% as a food waste ratio resided in the range of 20 to 40% suggested by the existing literature [18]. For S7, the community partner’s datasets suggested that 17.2% of received food surplus is spoiled or wasted during redistribution. S6 had a higher quality of donated food surplus, which produced 30% less food waste than S5. Similarly, S8 had 30% less food waste than S7 due to the improved food quality. The uncertainty rationale summarization was listed in Table S6.

According to the investigation at the food bank, the portions of food waste transported to pig farms and landfills were 60% and 40%, respectively. The same ratios were applied in intermediary organizations’ management options for fair comparison. The amount of food waste sent to the landfilling facility and the amount of food waste sent to the pig farm are provided in Table S1. The average distances among donors, food banks, intermediary organizations, and food pantries were calculated using geolocations in Google Maps. Based upon the consultation with local stakeholders, there was a major difference in food waste treatment methods among donors. Some donors often adopted composting practices for food waste treatment (donor-composting), while other donors heavily relied on the landfill for handling their food waste (donor-landfilling). In 2022, 36.7% of surplus food ended up in landfills, while 18.3% was directed to composting [30].

The background life cycle inventory was primarily compiled by utilizing the ecoinvent version 3.6 database [28]. The unit life cycle environmental impacts associated with composting, transportation, food production of individual food types, and pig meal were estimated based on the ecoinvent database. The life cycle impacts of on-site composting were computed based on the weight of food waste for composting and the unit impact of the composting process in the ecoinvent database. A diesel-powered truck with a carrying capacity of 3.5–4 tons and a refrigerator was used to transport the food surplus. The life cycle impacts of transportation were determined by the weight of food surplus and transportation distances. The life cycle impacts of producing 1 kg of apple, banana, grape, lemon, lime, mango, melon, orange, peach, pineapple, strawberry, sweet beet, bell pepper cabbage, carrot, cauliflower, celery, corn, cucumber, eggplant, spinach, lettuce, onion, potato, tomato, and zucchini were extracted from the ecoinvent database. The life cycle impacts of other products were obtained from the literature [31,32,33,34,35,36,37,38,39]. The avoided environmental impacts of food production due to food donation were estimated based on the weight of distributed food surplus and the unit impact of food production. The avoided environmental impacts of pig meal production due to using food waste as pig feed were estimated by the weight of food waste as pig feed and the unit impact of soybean meal as pig feed, since soybean meal is frequently used as pig feed [40]. Overall, the life cycle inventory was compiled and computed using OpenLCA version 1.10.3 [41], in agreement with the ISO standard for LCA.

### 2.3. Life Cycle Impact Assessment (LCIA)

In the LCIA phase, the inventories of emissions were classified into categories to evaluate the impacts on the environment. As shown in Table S3, this study focused on cumulative energy demand (CED), water use, global warming potential (GWP), eutrophication potential (EU), and acidification potential (AD) over the supply system, as covered in the existing literature. The CED method calculates the cumulative energy demand, which equals the total fossil fuel usage. Water use was estimated following the terms of the blue water footprint guidelines [42]. The IPCC method was adopted to calculate the GWP over a 100-year time frame. The Tool for Reduction and Assessment of Chemicals and other Environmental Impacts (TRACI) model version 2.1, developed by the US EPA, was used to calculate the life cycle of AD and EU. TRACI is the only tool that provides North America-specific characterization factors [43].

### 2.4. Life Cycle Interpretation

A scenario analysis was performed to identify the most environmentally friendly supply chain configurations and operational practices. The stage contribution analysis describes the relative influences of each stage on life cycle environmental impacts. Understanding the impacts of each stage aids in suggesting effective mitigation strategies.

The sensitivity analysis in this paper aims to understand the relative influences of the input foreground on the overall environmental impacts. The sensitivity analysis provides insights into model uncertainty and provides directions for future data collection. This study used the one-at-a-time technique for sensitivity assessment [44]. By perturbing each parameter while holding other parameters constant, the sensitivity range for individual parameters can be calculated to determine its influence on the life cycle impact results. The tested input parameters included the allocation proportion of food waste for landfilling and pig farms, life cycle impacts of food production for the top ten contributing food types, life cycle impacts of transportation, life cycle impacts of composting, and route distance. In total, 15 input parameters were tested for each environmental impact category. As an example, the 15 input parameters for the sensitivity analyses of the GWP impact are listed in Table 3. Additional parameters in the sensitivity analysis of EU and AD are listed in Table S4. The sensitivity ranges of the GWP intensity are the intervals bounded by the 5th and 95th percentile obtained from the ecoinvent database.

Additionally, a further sensitivity analysis was conducted to evaluate the influence of avoided food production on the environmental net savings resulting from food surplus donations. Most of the preceding LCA models operated under the assumption that food surplus donations completely obviate the necessity for new food production [14,15,18,20,21]. Only one study [19] has included scenarios considering both 100% and 0% avoidance of new food production through surplus food donation. Nevertheless, no study has proven this assumption yet. In our analysis, we explored the environmental net benefits for both donor-composting and donor-landfilling under scenarios where food surplus donations are presumed to avert 0%, 50%, and 100% of new food production.

## 3. Results

### 3.1. Comparing Environmental Impacts of Redistribution Scenarios

**Figure 2:**
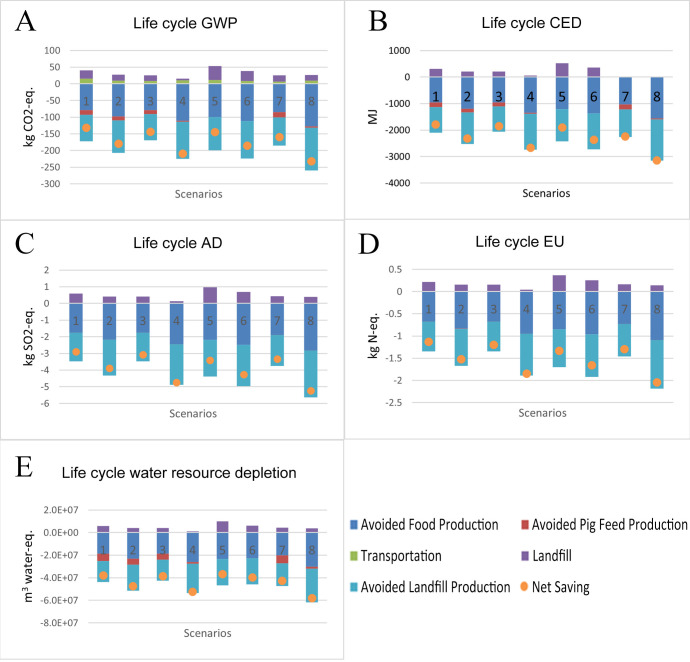
(**A**–**E**). Life cycle environmental impacts of eight donation systems with donors landfilling the food waste for GWP (**A**), CED (**B**), AD (**C**), EU (**D**), and water source depletion (**E**).

As shown in Figure 2, all scenarios realized net life cycle environmental benefits. Figure 2A–E correspond to the life cycle GWP, CED, EU, AD, and water resource depletion of surplus food redistribution scenarios for donor-landfilling, respectively. Among the eight scenarios, the net savings of life cycle GWP ranged from 132 to 233 kg CO_2_-eq. The net savings of life cycle AD had a range from 2.30 to 5.24 kg SO_2_-eq. The life cycle EU obtained net savings ranging from 1.13 to 2.04 kg N-eq. The net savings of life cycle CED spanned from 1791 to 3140 MJ. The range of net savings from 3.7 × 10^7^ to 5.8 × 10^7^ m^3^ was estimated for water resource depletion. Overall, net savings were observed for life cycle GWP, AD, EU, CED, and water use across all scenarios.

Regardless of centralized or decentralized supply chain configurations, operational improvements can significantly enlarge life cycle environmental benefits (as shown in Figure 2). For the centralized supply chain configurations, S2, S3, and S4 with operational improvements achieved higher net savings than S1 for all life cycle impacts. Employing additional sorters at the food bank for S2 resulted in 25–37% more net savings than S1 for life cycle GWP, CED, AD, EU, and water depletion, respectively. When the food quality from the donors improved for S3, 1.6–9% more life cycle environmental benefits were realized than S1, due to less food waste during redistribution. Since S4 employed additional sorters and improved the quality of food surplus, S4 showed 38–63% more net savings for all life cycle impacts than S1. Similarly, for the decentralized supply chain configurations, the improved food quality led to increased life cycle environmental benefits. S6 showed 8–25% more net savings than S5. S8 had 35% to 57% more net savings than S7.

The decentralized supply chain configuration did not always perform better than the centralized supply chain configuration. The majority of decentralized scenarios presented higher magnitudes of environmental benefits than the status quo centralized supply chain configuration (S1). However, three decentralized scenarios (S5, S6, and S7) showed smaller environmental benefits than the centralized supply chain configuration with additional sorters and high-quality donations (S4).

Overall, S8 presented the greatest magnitudes of environmental benefits among all eight scenarios for all environmental impact categories (Figure 2). In contrast, S1 or S5 showed the smallest magnitudes of environmental benefits. S1 ranked as the worst scenario for life cycle global warming, eutrophication, acidification, and CED. S5 ranked as the worst scenario for life cycle water depletion, with a value 3% smaller than S1.

### 3.2. Comparing Environmental Impacts of Donors’ Waste Handling Practices

**Figure 3:**
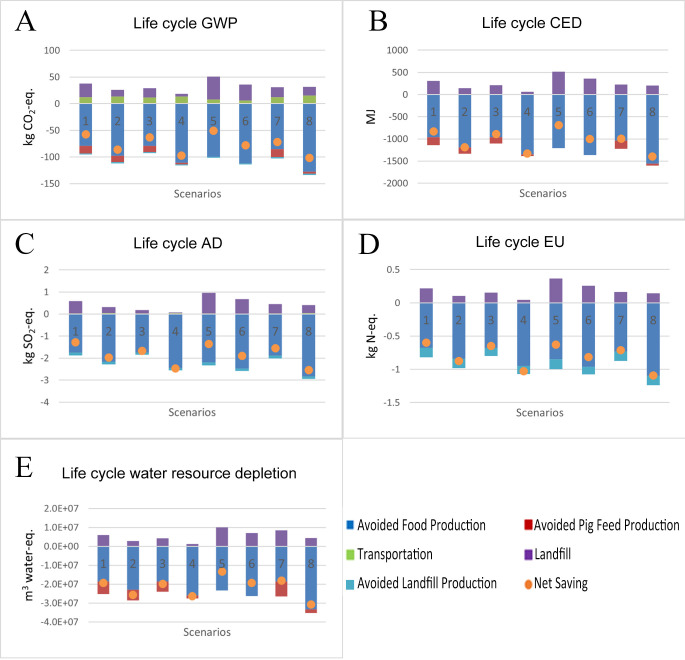
(**A**–**E**). Life cycle environmental impacts of eight donation systems with donors composting the food waste for GWP (**A**), CED (**B**), AD (**C**), EU (**D**), and water source depletion (**E**).

Figure 3 exhibits the life cycle assessment result for the food donation system associated with donor-composting. The comparison between the donor-composting and donor-landfilling scenarios suggested that the environmental benefits of donor-landfilling scenarios were higher than those of donor-composting for the same supply chain configurations and operational practices. For example, switching from donor-composting to donor-landfilling for scenario (S1) led to an increase in the net GWP savings ranging from 58 to 258 kg CO_2_-eq. Similarly, approximately 127%, 89%, 115%, and 96% increases were observed for life cycle AD, EU, CED, and water resource depletion, respectively. On average, switching from donor-composting to donor-landfilling increased the net savings of GWP, AD, EU, CED, and water resource depletion by 45%, 42%, 38%, 44%, and 40%, respectively. The trends among various supply chain configurations and operational management scenarios associated with donor-composting are consistent with donor-landfilling. Among all scenarios, S8 and S4 remained as the best scenarios, presenting the highest magnitudes of life cycle environmental benefits. In contrast, S1 and S5 ranked as the worst scenarios, showing the lowest magnitudes of life cycle environmental benefits.

### 3.3. Comparing Environmental Impacts of Life Cycle Stages

As shown in Figure 2, featuring donor-landfilling scenarios, landfilling the food waste during the redistribution of food surplus and transporting food surplus resulted in environmental damage. In contrast, the avoided agricultural production, landfilling, and pig feed due to donation efforts offset environmental damages. Landfilling food waste had the largest share of environmental damage, which occupied over 90% of the environmental damage for all life cycle impacts. Transportation resulted in the remaining 10% of environmental damage. The total amount of damage caused by the landfilling and transportation stages was completely offset by the avoided activities. The avoided food production stage and avoided landfilling due to donation contributed to about 45% and 50% of the total negated damages, respectively. Additionally, the avoided pig feed production negates less than 10% of total life cycle impacts.

As shown in Figure 3, for donor-composting scenarios, landfilling and transporting food surplus caused environmental damage. On the contrary, avoiding the production of fresh produce, pig feed, and composting by donation provided environmental benefits. Landfilling food waste contributed to a considerable share of environmental damage, which occupied 42% to 84% of the environmental damage for all life cycle impacts. Transportation caused the rest of the environmental damage. The environmental damage caused by the landfilling and transportation stages was entirely offset by avoiding the production of fresh produce, pig feed, and composting. The avoided food production and composting together contributed to about 80% and 90% of total environmental benefits. Additionally, the avoided pig feed production generated the remaining (around 10–20%) total life cycle environmental benefits.

### 3.4. Sensitivity Analysis

**Figure 4:**
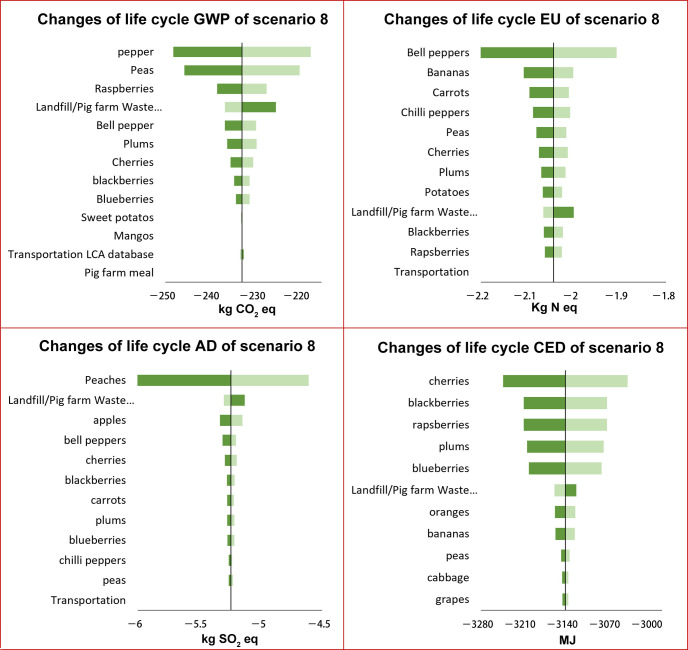
(**A**–**D**). Sensitivity analysis of scenario 8 for donation system with donors landfilling food waste in terms of GWP (**A**), EU (**B**), AD (**C**), and CED (**D**).

**Figure 5:**
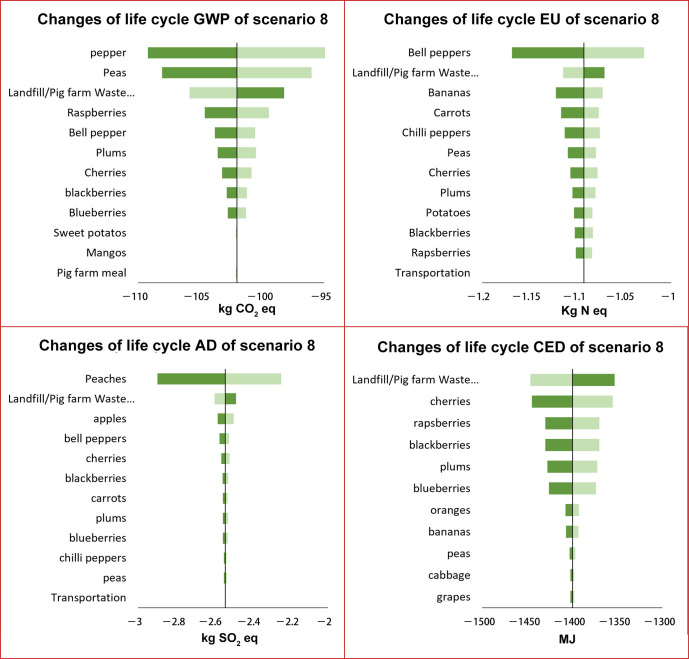
(**A**–**D**). Sensitivity analysis of scenario 8 for donation system with donors composting food waste in terms of GWP (**A**), EU (**B**), AD (**C**), and CED (**D**).

As displayed in Figure 4 and Figure 5, the sensitivity of input parameters varied significantly across different environmental impact categories. For the global warming impact of scenario 8A, the GWP intensities of peppers, peas, and raspberries ranked as the top three influential factors. The global warming impact varied by 6.8%, while the global warming intensity of peppers varied from −248.2 to −217.4 kg CO_2_ eq. Following these three factors, the landfill/pig meal fraction also presented considerable influences on the GWP. The rest of the input parameters such as other produce, transportation distances, and pig meal had minimal impacts (less than 0.1%). For the EU, the EU intensities of bell peppers, bananas, carrots, chili peppers, and peas were identified as the top five influential factors. The other input parameters have a minimal impact. For AD, The AD intensity of peach production was the driving factor for the sensitivity of the total life cycle AD impact. Varying the AD intensity of peach production from its 5th to 95th values could lead to life cycle AD varying by ±15%. Varying the remaining factors led to less than 1% of changes in the life cycle of AD. The top influential factors for CED were the CED intensities of producing cherries, blackberries, and raspberries. Following these three, the landfill/pig meal fraction and the production of plums and blueberries also had a comparable impact on the CED. The rest of the parameters had negligible impacts.

The top three factors with the greatest influence on the environmental impact were consistently identified across most scenarios, except for the landfill/pig meal fraction. The life cycle impact densities of fresh produce consistently ranked as the top influential parameter. The landfill/pig meal fraction ratios ranked higher for S8 than S4 for GWP, CED, AD, and EU impacts (Figures S1 and S2), indicating that the landfill/pig meal fraction was more influential for S8 than for S4.

The top influential factors of donor-composting and donor-landfilling were generally consistent for a given scenario and impact category, except for CED in scenario 8. For this impact category in scenario 8B, the CED impact varied by +7% and −3% when the CED intensity landfill/pig meal was varied from −1447 to −1353, making it the most influential factor instead of cherry production.

The maximum environmental benefits are yielded when surplus food donations entirely prevent new food production. For non-farm donors, food surplus donation results in significant net savings in GWP (232.8 kg CO_2_ eq.), CED (3140.1 MJ), AD (5.2 kg SO_2_-eq.), and EU (2.0 kg N-eq.) when new food production is fully avoided (Table S5). However, these benefits are substantially reduced when surplus food donations fail to completely offset new food production. For instance, if surplus food donations fail to prevent any new food production, environmental savings can decrease by up to 55% compared to scenarios achieving a 100% offset. Nevertheless, even in cases where surplus food donations from non-farm donors do not eliminate any new food production, environmental net savings are still generated. For farm donors, successfully preventing 100% of new food production leads to notable net savings in GWP, CED, AD, and EU, amounting to 102.0 kg CO_2_ eq., 1399.8 MJ, 2.5 kg SO_2_-eq., and 1.1 kg N-eq., respectively. However, reducing the effectiveness of surplus food donations in preventing new food production to 50% results in a reduction in environmental net savings by up to 63%. If donations cannot avoid any new food production, food surplus donations from farm donors exhibit net burdens in GWP, CED, and AD, but with no significant impact on EU.

## 4. Discussion

### 4.1. The Impacts of Redistribution Strategies and Donor’s Waste Management Practices

Rescuing food surplus presented significant environmental benefits under the modeled assumptions of this study. Although redistributing food surplus from donors to recipients requires transportation and generates food waste due to food spoilage and expiration, the environmental damages associated with food donation chains are totally offset by the avoided landfilling and food production. Both landfilling and food production are resource-intensive activities and result in global warming, eutrophication, and acidification impacts. Avoiding landfilling surplus food and offsetting food production yield high magnitudes of environmental benefits. This aligns with findings from earlier studies, highlighting that the prevention of new food production stands as a significant factor contributing to the environmental advantages of surplus food donation [14,16,21]. Moreover, evading food waste disposal has been shown to yield beneficial outcomes [45]. Damiani et al. [15] and Sulis et al. [19] identified both avoided landfilling and avoided food production as key contributors to the environmental benefits of surplus food donation. In contrast, Albizzati et al. [14] and Sundin et al. [18] focused on the benefits of avoided food production in their food donation LCA studies, without accounting for landfilling. In this study, even without considering the benefits of avoiding landfilling, S8 still demonstrates the highest environmental benefits across all impact categories compared to the other eight scenarios. As demonstrated in this study, all eight redistribution scenarios, regardless of supply chain configurations and operational management options, presented significant environmental benefits for reducing energy/water use and mitigating global warming, eutrophication, and acidification impacts.

Our model results indicate that increasing the sorting capacities of food banks, improving the quality of donated food, and altering the supply chain configurations can increase the net environmental benefits of food rescue. First, reducing food waste during redistribution by adding sorters and receiving high quality donations can increase the environmental benefits of food donation systems. This study suggests that adding more produce sorters at the food bank achieves a significant improvement in all life cycle impacts. Having additional sorters enables the recovery of more surplus food from landfills for redistribution and consumption. In this study, we assumed that the redistributing or reuse of surplus food can avoid the production of new food. When the amount of avoided food production expands (S2 and S4 are better than S1), the net benefits of life cycle impacts increase. Receiving high-quality food from donors can reduce the amount of waste generated during redistribution, consequently improving the environmental performance of food donation systems (S3 and S4 are better than S1; S6 is better than S5; S8 is better than S7). Second, using food waste for pig meal rather than landfilling food waste can increase environmental benefits (S8 is better than S6). As discussed in the previous paragraph, avoiding landfilling surplus food and offsetting pig meal production have substantial environmental benefits. This is consistent with the US EPA’s waste management scale in which source reuse is preferred over waste treatment and disposal. In some areas (e.g. Sweden), the landfilling of organic waste is illegal [17], which forces food waste to be reused or recycled. Third, directly transporting surplus food from donors to pantries reduced transportation distances, resulting in a small reduction in environmental releases (S5 and S6 are better than S1). However, it is worth noting that decentralized distributions do not always outperform centralized distributions (S4 is better than S6 but worse than S8). Although decentralized options tend to have shorter transportation, reducing food waste during redistribution and diverting food waste from landfills to animal farms yielded higher benefits than shortening distances (S4 is better than S6). Overall, the scenarios which rescue the largest amount of surplus food and generate the least amount of food waste had the highest environmental benefits.

Finally, the food donation system associated with donor-landfilling gains more net benefits than donor-composting under the modeled assumptions of this study. These differences caused by donor types resulted from the different waste management approaches adopted by donor-composting and donor-landfilling. Donor-composting uses composting rather than landfilling their food waste. Avoiding landfilling will result in larger benefits than avoiding composting. Rescuing food from donor-landfilling showed slightly higher environmental benefits than donor-composting.

### 4.2. Strategies Capable of Enlarging Environmental Benefits of Food Donation Systems

All studies reviewed in preparation for this analysis indicated the environmental benefits of food donation. Although there are large disparities in study scopes and methods, all studies recognized that food surplus donation has more benefits than burdens. All studies reported that food donation-related GWP reductions ranged from 0.35 to 5 kg CO_2_ eq/kg food donated [14,15,16,17,18,19,20,21]. In this study, compared to the baseline food donation strategy, improving operational management and altering supply chain configurations resulted in GWP reductions ranging from 0.33 to 0.69 kg CO_2_ eq/kg redistributed food surplus.

This study represents the first study assessing how the quality of donated food, sorting capabilities, and distribution logistics affect the environmental impacts of food donation systems. Improving sorting capacity and improving food quality both led to a significant reduction in food waste and ultimately mitigated environmental impacts. In this study, S8 had the largest net savings in all the five impact categories because S8 had the smallest food waste due to the high donation quality and low transportation distance. Despite focusing on different scenarios, Albizzati et al. [14] and Sulis et al.’s [19] studies also found that the scenario of achieving the largest reduction in food waste tended to present the largest net environmental savings. Additionally, reducing food transportation distances can slightly decrease the environmental burdens associated with donated food redistribution processes. Previous studies have also identified the transportation of food surplus as a burden in assessing the environmental impacts of food surplus management systems [15,16,18,21].

This study revealed that optimal food surplus distribution strategies generated net savings in all five environmental impact categories. Adopting the best scenario (S8) can provide at least 70% more environmental benefits than the status quo scenario (S1) for the GWP, AD, EU, and CED categories. Utilizing the best scenario (S8) can offer around 40% more environmental benefits than the status quo scenario (S1) for the water depletion category. Therefore, it is important to recognize the environmental benefits of food donation systems beyond the savings of energy use and greenhouse gas emissions.

This study examined the environmental impacts of different donated food types through a sensitivity analysis. The results indicated that food types can significantly affect environmental sensitivity, with certain foods having greater impacts on specific categories, such as GWP, EU, AD, and CED. Notably, some of the highest-impact foods were peppers, peas, bell peppers, peaches, and cherries. These findings emphasize the importance of considering the environmental consequences of food rescue efforts and the potential benefits of prioritizing the rescue of high-impact foods.

### 4.3. Study Strengths and Limitations

This study presents several merits regarding study scope and data collection. First, this is one of the first studies to compare various redistribution scenarios of food donation systems. This comparison reveals the environmental benefits of food donation and redistribution systems and also aids in the design of the redistribution chains to maximize environmental benefits. Second, the study relied on the first-hand datasets collected from a local food bank and contains details such as the weight of redistributed food surplus for 43 specific food types, the weight of food waste generated during redistribution, and the fractions of food waste sent to landfills and pig farms, respectively. Last, this study assessed various life cycle impacts including energy use, water depletion, global warming, eutrophication, and acidification.

This study is limited in capturing spatial and temporal dynamics, behavioral re-bound effects, and labor and cost trade-offs. First, this study only took place in the Capital Region, and the findings are not necessarily generalizable to other regions and food systems. Due to resource constraints, the authors were only able to volunteer and gather real datasets at the food bank for two weeks in 2019. This short time frame could not fully reflect the temporal dynamics of food donation and redistribution systems, such as storage, spoilage, seasonal effects, donor mix, or long-term operational dynamics. Further studies are needed to investigate how the results might transfer to rural areas, different logistics infrastructures, or non-U.S. contexts. Second, this study did not include the building maintenance and packaging requirements of emergency food organizations. For a fair comparison, the same amount of food surplus was available at donors for emergency food organizations among eight scenarios. We assumed the same packaging requirements for all scenarios since we did not have datasets regarding how emergency organizations handle packaging. Long-term data collection is recommended to reflect the temporal dynamics and different packaging handling practices. Third, the effects of food rescue on food production are uncertain. This study performed sensitivity analyses to understand the influences of possibly avoided food production on the overall environmental impacts of food rescue and redistribution. Though the one-at-a-time sensitivity analysis does not capture parameter interactions, there are no known or logical potential interactions among the tested parameters in this study. Fourth, labor inputs, costs, and workforce constraints are not assessed in this study, which may limit real-world feasibility interpretation. We encourage future studies connecting LCAs with economic models to explore the interactions between food rescue, food production, and economic impacts. Additionally, the study did not consider the rebound effects. Future studies on connecting LCAs with social-behavioral models may reveal the rebound effects. Furthermore, due to the data’s availability and relevance, this study specifically centers the focus on vegetables and fruits. More community-engaged studies are needed on the environmental impacts of more diverse fruits and vegetables in donation systems.

### 4.4. Implications for Food Waste Management/Food Donation

All scenarios regardless of supply chain configurations and operational management options showed significant life cycle environmental benefits for GWP, AD, EU, and the use of energy and water resources in this study. This proves that rescuing food surplus is a preferred method for food surplus management, as mentioned in the waste management hierarchy by the US government. To achieve the goal of sustainability, rescuing food surplus should be emphasized and supported by policies.

Adding sorters and receiving high-quality donations are capable of greatly increasing the environmental benefits of the food rescue system. This indicates that proper improvement strategies can further increase the environmental benefits of food rescue systems. The strategies for promoting high-quality donations and increasing sorting capacity are suggested to enhance the environmental benefits of the food rescue system. In the meantime, we should be aware that adding sorters may increase the labor costs in the food bank and connecting organizations. Moreover, the waste generated from the food rescue system cannot be completely avoided. Reusing this waste for pig meal rather than landfilling can increase the environmental benefits. Connecting food rescue organizations with animal farms or composting facilities for coordinated food waste management can achieve larger systemic benefits. Overall, these findings provide valuable information for aiding in policy development and decision-making in the areas of food rescue and waste management.

## 5. Conclusions

This LCA of the food donation system in the Capital Region in New York State suggested that the food donation system presented environmental benefits across energy use, water use, global warming, acidification, and eutrophication categories. The comparative results clearly indicated that S8, in which intermediary organizations collected and gleaned the surplus food from donors and then directly transported it to food pantries, had the largest environmental benefits for all evaluated categories. The comparison showed that improving the food quality of donated surplus food, adding more sorters in food banks or food pantries, and reducing transportation distances for food donation and redistribution can improve the environmental benefits of food donation systems. These scenario comparisons provided foundational knowledge for possible improvement strategies for food donation systems. These findings offer some of the first quantifiable data on the environmental benefits of recovering and redistributing surplus produce to assist policymakers in quantitatively understanding the potential environmental advantages of initiatives designed to reduce food waste. However, the most environmentally preferred strategies may not always be operationally feasible or economically effective. Further studies are needed to find optimal strategies which are environmentally sustainable, operationally feasible, and cost effective.

## Figures and Tables

**Table 1:** Eight representative scenarios reflecting different supply chain configurations and operational management options at Regional Food Bank.

Scenario #	Description	Supply Chain Configurations	Operational Management Options		
			Sorting Capacity	Quality of Donated Food	Food Waste Management
1	Food Donor to Food bank to Food Pantry	Centralized distribution	1 sorter	Status quo food quality	Landfill and pig farm
2	Food Donor to Food bank to Food Pantry	Centralized distribution	2 or more sorters	Status quo food quality	Landfill and pig farm
3	Food Donor to Food bank to Food Pantry	Centralized distribution	1 sorter	Improved food quality	Landfill and pig farm
4	Food Donor to Food bank to Food Pantry	Centralized distribution	2 or more sorters	Improved food quality	Landfill and pig farm
5	Food Donor to Food Pantry	Decentralized distribution	1 sorter	Status quo food quality	Landfill
6	Food Donor to Food Pantry	Decentralized distribution	1 sorter	Improved food quality	Landfill
7	Food Donor to Intermediary to Food Pantry	Decentralized distribution	1 sorter	Status quo food quality	Landfill and pig farm
8	Food Donor to Intermediary to Food Pantry	Decentralized distribution	1 sorter	Improved food quality	Landfill and pig farm

**Table 2:** Fruit and vegetable types distributed by food bank and weight fraction for each type.

Fruits	Fraction	Vegetables	Fraction
apples	12.51%	beets	3.31%
bananas	10.43%	bell peppers	4.14%
blackberries	4.17%	broccoli	2.21%
blueberries	3.65%	cabbage	11.05%
cherries	6.26%	carrots	13.26%
grapes	9.38%	cauliflower	2.21%
lemons	2.09%	celery	4.42%
limes	2.09%	chili peppers	1.10%
mangoes	2.50%	collards	2.21%
melon (cantaloupes)	5.21%	corn	4.14%
oranges	15.64%	cucumber	2.76%
peaches	4.17%	eggplant	1.66%
pineapples	2.61%	green leaves/spinach	2.76%
plums	4.17%	green squash	1.10%
raspberries	4.17%	kale	0.86%
strawberries	8.34%	lettuce	2.76%
watermelon	2.61%	mushrooms	2.49%
		onions	5.52%
		peas	3.31%
		peppers	3.31%
		potatoes	7.73%
		sweet peas	3.31%
		sweet potatoes	3.31%
		tomatoes	5.52%
		yellow squash	2.76%
		zucchini	2.76%

**Table 3:** Sensitivity test.

Parameters	Unit	Current	Sensitivity Range	
% surplus food sent to landfill	%	0.4	0.2	0.6
% surplus food sent to pig farm	%	0.6	0.8	0.4
Transportation distance	(km)	76.92	22.06	447.33
GWP intensity of pepper	(kgCO_2_/kg)	4.7	3.29	6.11
GWP intensity of peas	(kgCO_2_/kg)	3.94	2.758	5.122
GWP intensity of raspberries	(kgCO_2_/kg)	1.34	0.938	1.742
GWP intensity of bell peppers	(kgCO_2_/kg)	4.7	3.29	6.11
GWP intensity of plums	(kgCO_2_/kg)	0.8	0.56	1.04
GWP intensity of cherries	(kgCO_2_/kg)	0.41	0.287	0.533
GWP intensity of black berries	(kgCO_2_/kg)	0.424	0.2968	0.5512
GWP intensity of blue berries	(kgCO_2_/kg)	0.44	0.308	0.572
GWP intensity of sweet potato	(kgCO_2_/kg)	0.45	0.0259	0.0446
GWP intensity of mangos	(kgCO_2_/kg)	0.4556	0.0156	0.0262
GWP intensity of transportation	(kgCO_2_/tkm)	0.41947	0.07436	0.246915
GWP intensity of composting	(kg CO_2_/kg)	0.00581	0.00447	0.00746

Note: Table includes the top ten contributing food types out of a total of 43 types for the total life cycle environmental impacts.

## Data Availability

The original contributions presented in the study are included in the article and Supplementary Materials; further inquiries can be directed to the corresponding author.

## Supplementary Materials

The following supporting information can be downloaded at https://www.mdpi.com/article/10.3390/foods15040645/s1. Figure S1: Sensitivity analysis of scenario 4, for donation system with donors landfilling food waste; Figure S2: Sensitivity analysis of scenario 4, for donation system with donors composting food waste; Table S1: Life cycle inventory of background process; Table S2: Parameters for foreground life cycle inventory; Table S3: Life cycle assessment method; Table S4: Additional parameters in sensitivity analysis; Table S5: Sensitivity test for the net savings with respect to the percentage of avoided new food production. Table S6: Uncertainty rationale summarization. References [28,31,32,33,34,35,36,37,38,39] are cited in the supplementary materials.
