# Supplementary material for: Comparing the Environmental Impacts of Representative Food Donation and Redistribution Strategies
Source: Foods. 2026 Feb 11;15(4):645. doi: 10.3390/foods15040645 (PMC12939375; doi:10.3390/foods15040645)
Supplement: Supplementary file 1 [file foods-15-00645-s001.zip › foods-4075157-supplementary.pdf]

Supplemental Information for:

Comparing the environmental impacts of representative food donation and redistribution strategies

This SI includes detailed information regarding processes, parameters and models used for generating life cycle inventory and impact assessment as well as sensitivity assessment. This file contains:

Tables: 6
Figures: 2
Pages: 5

Table S1 Life cycle inventory of background process

Process	Data sources
Food production for Apple, banana, grape, lemon, mango, melon, orange, peach, pineapple, strawberry, sweet beet, bell pepper, cabbage, carrot, cauliflower, celery, collard, corn, cucumber, eggplant, spinach, lettuce, onion, potato, tomato, and zucchini	Ecoinvent (Wernet et al., 2016)
Food production for Blackberry, blueberry, cherry, limes, plum, raspberry, watermelon, collard, kale, mushroom, pea, sweet pea	Literature (Audsley et al., 2010; FleishmanHillard, 2016; Kim & Dale, 2004; Maraseni et al., 2010; Peano et al., 2015; Pergola et al., 2013; Svanes & Johnsen, 2019; Tassielli et al., 2018; Yuttitham, 2019)
Transportation	Ecoinvent (Wernet et al., 2016)
composting	Ecoinvent (Wernet et al., 2016)
pig farm meal	Ecoinvent (Wernet et al., 2016)

Table S2 Parameters for foreground Life cycle inventory

Parameters	Values/Ranges	Data sources	Year
The amount of received food donation at the food bank (Kg)	391.8	Measured at Regional food bank	2019
The amount of food surplus redistributed from the food bank (Kg)	217.7	Measured at Regional food bank	2019
Transportation Distance (parameter)	13.71-277.96	Calculated based on geolocations of food donors and recipients	N/A
The percentage of surplus food sent to landfill	40	Regional food bank	2019
The percentage of surplus food sent to pig farm	60	Regional food bank	2019

Table S3 Life cycle assessment method

Names	description	Data sources	Year
**IPCC**	IPCC 2013 GWP 100a	OpenLCA	2020
**CED**	Cumulative Energy Demand	OpenLCA	2020
**TRACI**	TRACI2.1	OpenLCA	2020

Table S4 Additional parameters in sensitivity analysis

**Parameters**	**Unit**	**Mean/Median**	**Sensitivity**	**range**
EU intensity of transportation	(Kg N eq./tkm)	0.00006373	0.0000612	0.00006625
AD intensity of transportation	(Kg SO_2 eq./tkm)	0.055	0.055	0.06
EU intensity of composting	(Kg N eq./Kg)	0.000032	0.000025	0.00004
AD intensity of composting	(Kg SO_2 eq./Kg)	0.017	0.012	0.022

Table S5 Sensitivity test for the net savings with respect to the percentage of avoided new food production.

	**Non-farm donors**			**Farm donors**		
**Avoided new food production**	100%	50%	0%	100%	50%	0%
GW	-232.8	-168.9	-105.1	-102.0	-38.1	25.7
CED	-3140.1	-2362.7	-1585.3	-1399.8	-622.5	154.9
AD	-5.2	-3.8	-2.4	-2.5	-1.1	0.3
EU	-2.0	-1.5	-0.9	-1.1	-0.5	0.0

Table S6 Uncertainty rationale summarization.

Assumption	Sources/ references
30% waste reduction with additional sorters or higher quality donations	Provided by food bank employee, based on their internal evaluation, and backed up literature
Excluding packaging/cold-storage energy	No data availability

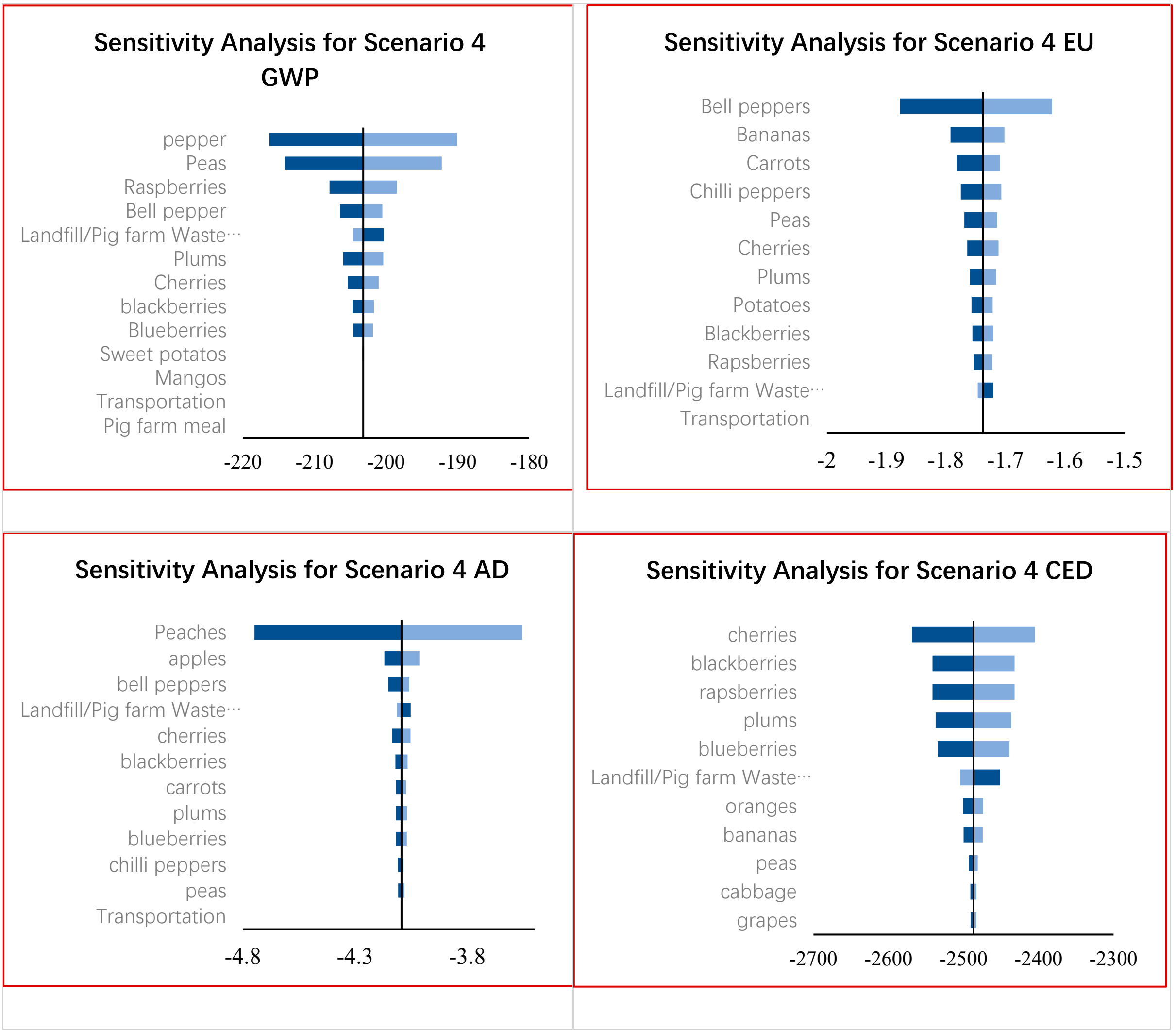

Figure S1 Sensitivity analysis of scenario 4, for donation system with donors landfilling food waste.

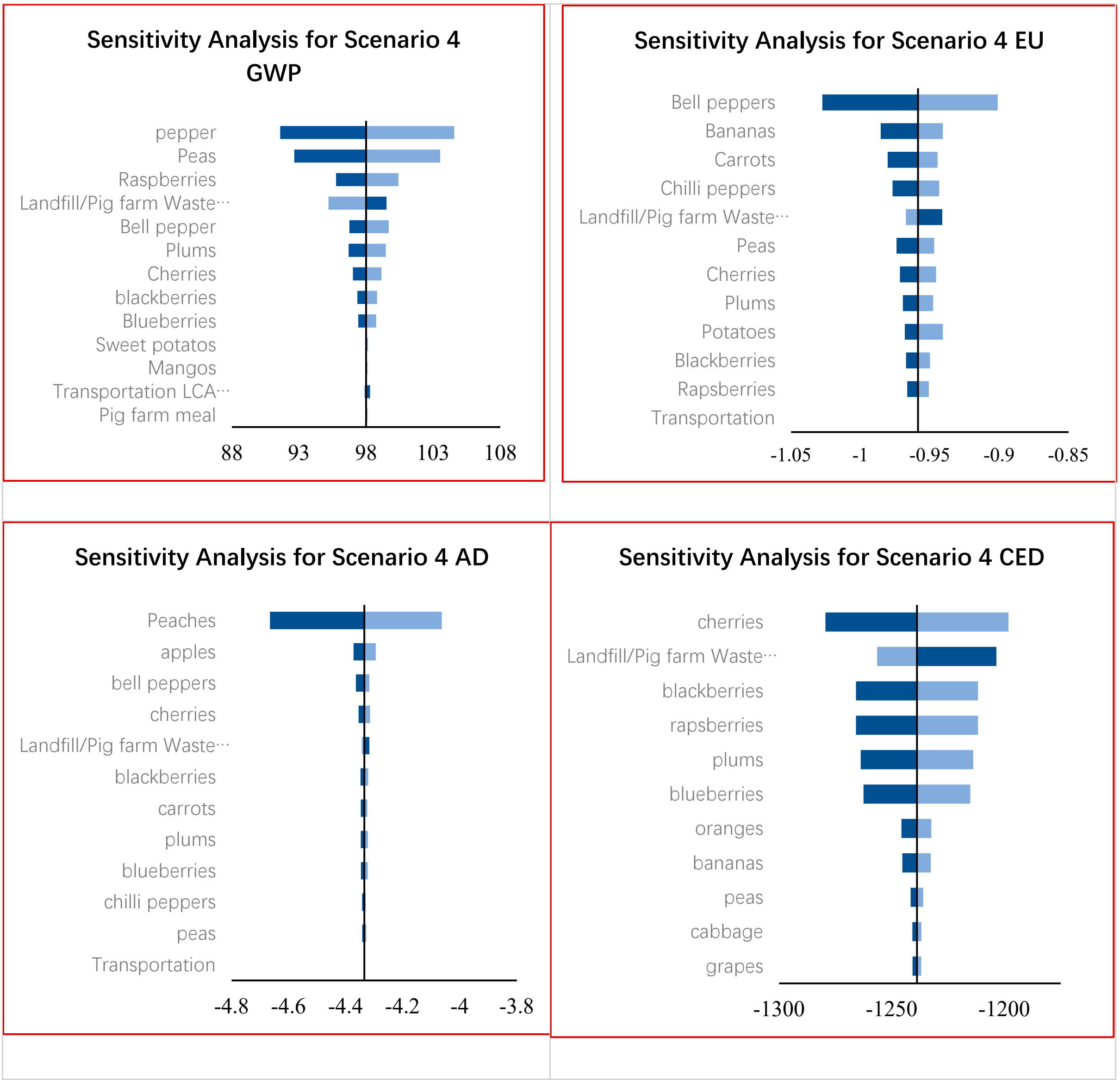

Figure S2 Sensitivity analysis of scenario 4, for donation system with donors composting food waste.
